# The efficacy and safety of polydeoxyribonucleotide for the treatment of knee osteoarthritis: Systematic review and meta-analysis of randomized controlled trials

**DOI:** 10.1097/MD.0000000000017386

**Published:** 2019-09-27

**Authors:** Man Soo Kim, Ryu Kyoung Cho, Yong In

**Affiliations:** Department of Orthopaedic Surgery, Seoul St. Mary's Hospital, College of Medicine, The Catholic University of Korea, Seoul, Korea.

**Keywords:** injection, meta-analysis, osteoarthritis, polydeoxyribonucleotide, randomized controlled trial

## Abstract

**Introduction:**

The purpose of this study was to use meta-analysis techniques to evaluate the efficacy and safety of polydeoxyribonucleotide (PDRN) injections for knee osteoarthritis (OA) treatment.

**Methods:**

Multiple comprehensive databases, including MEDLINE, EMBASE, and the Cochrane Library, were searched in November 2018 for studies that compared the effectiveness and safety of intra-articular PDRN injection for the knee joint with hyaluronic acid (HA) injection. Two reviewers independently determined study inclusion and they extracted data using a standardized data extraction form. The predefined primary outcome was Visual Analogue Scale. Secondary outcomes included Knee Injury and Osteoarthritis Outcome Score (KOOS), Knee Society Score (KSS), and adverse events.

**Results:**

Five randomized controlled trials were included in the meta-analysis. After 1 and 2 months, patients in the PDRN group showed significantly better improvement in pain than the HA group (*P* = .04 and *P* = .02, respectively). There was no significant difference in pain after 4 months. The pooled analysis showed that no significant differences were seen in function (KOOS and KSS) scores between the PDRN and HA groups (all *P* > .05) at all time points. There was no significant difference in adverse events between 2 groups (relative risks = 2.15, 95% confidential interval: 0.17–26.67, *P* = .55).

**Conclusion:**

The intra-articular use of PDRN was similar in function to HA, and the pain-relief effect was superior to HA for 2 months post-injection. Therefore, it could be a favorable alternative to HA to treat persistent pain associated with knee OA while avoiding side effects.

**Level of evidence** I

## 1 Introduction

Knee osteoarthritis (OA), one of the most common age-related degenerative diseases, is marked by cartilage degeneration.^[1]^ It causes severe pain and loss of function, which can affect patient’ quality of life.^[2]^ Knee OA is a major cause of musculoskeletal disorders in elderly populations worldwide with a prevalence of 12% to 35%.^[3,4]^ Current conservative therapeutic approaches consist of analgesics, nonsteroidal anti-inflammatory drugs, and intra-articular corticosteroid and hyaluronic acid (HA) injections.^[5–7]^

Intra-articular HA injection is simple and commonly used to treat minor to moderate OA.^[8]^ HA can enhance the viscoelasticity of the synovial fluid, lubricate the articular surface, and reduce articular attrition, thereby protecting the cartilage from mechanical stress, maintaining elasticity, and relieving pain for a long time.^[9]^ Although HA's effectiveness has been demonstrated in several studies, it remains controversial.^[10]^

Polydeoxyribonucleotide (PDRN) is a complex of deoxyribonucleotide polymers of various chain lengths that is derived from trout or salmon sperm.^[11]^ PDRN was originally introduced as a therapeutic immune-stimulating agent to treat skin diseases, such as radio dermatitis, skin graft donor site, and photorefractive keratectomy.^[12]^ Polynucleotides are polymeric molecules capable of binding large amounts of water molecules.^[13,14]^ They also have the potential to reorganize surface cartilage structure by orienting and coordinating water molecules using 3-dimensional gel, which could provide deep moisture to joint surfaces when injected into joints.^[15]^ Gennero et al^[16]^ found that when PDRN was added to cultured chondrocytes in vitro, cell survival was higher than the control cells that were exposed to HA. Furthermore, degradation of proteoglycan, a representative extracellular matrix component of articular cartilage, showed decreased degradation in the PDRN-exposed cells. Gennero et al^[16]^ suggested that PDRN would be a better therapeutic agent than HA because of its enhanced ability to protect articular cartilage.

The purpose of this study was to use meta-analysis techniques to evaluate the efficacy and safety of PDRN injections for knee OA treatment. We hypothesized that PDRN injections would not be inferior to HA in terms of pain relief and functional improvement for treating patients with knee OA.

## 2 Methods

This study was performed following the guidelines of the Preferred Reporting Items for Systematic Reviews and Meta-Analysis (PRISMA) statement (S1 PRISMA Checklist).

### 2.1 Data and literature sources

The study design was performed according to the Cochrane Review Methods. Multiple comprehensive databases, including MEDLINE, EMBASE, and the Cochrane Library, were searched in November 2018 for studies in English that evaluated the effectiveness of intra-articular PDRN injection to the knee joint (S1 Search Strategy). There were no restrictions on publication year. Search terms included (Mesh “Osteoarthritis” and key words “arthritis,” “osteoarthritis,” “osteoarthrosis” “gonarthrosis” “gonoarthritis”), and (Mesh “polynucleotide” and key words “polynucleotide” “PDRN”). After the initial electronic search, manual searches of the reference lists and the bibliographies of identified articles, including relevant reviews and meta-analyses, were conducted to identify trials that the electronic search may have missed. Identified articles were individually assessed for inclusion. No ethic approval was necessary for this article because this study type was systematic review.

### 2.2 Study selection

Two reviewers independently determined study inclusion according to the predefined selection criteria. Titles and abstracts were screened for relevance. In cases of uncertainty, the full article was evaluated to determine eligibility. Discrepancies were adjusted through discussion. Level 1 randomized controlled trials (RCTs) with the following characteristics were included in the meta-analysis: population: patients diagnosed with knee OA; intervention: intra-articular injection with polynucleotide; comparison: intra-articular injection with HA; outcomes: values from at least one of the following scales—Visual Analogue Scale (VAS), Knee injury and Osteoarthritis Outcome Score (KOOS),^[17]^ Knee Society Score (KSS),^[18]^ or Western Ontario and McMaster Universities (WOMAC) osteoarthritis index,^[19]^ and adverse events, including local and systematic reactions. We excluded nonrandomized studies, retrospective studies, and articles for which we could not obtain the full text.

### 2.3 Data extraction

Two reviewers independently extracted data from each study using a standardized data extraction form. Disagreements were resolved by discussion, and those unresolved through discussion were reviewed by a third reviewer. The following variables were included: first author, publication year, country, number of participants, age, sex, body-mass index, intervention, and evaluation parameters. The predefined primary outcome was pain VAS score. Secondary outcomes included KOOS score, KSS total score, and adverse events. KOOS and KSS scores were evaluated for function in the meta-analysis. KOOS consisted of 5 patient-related issues: pain, other disease-specific symptoms, activity of daily living functions (ADL), sport/recreation functions, and knee-related quality of life (QOL). Among these 5 items, ADL was eliminated from the functional evaluations in our analyses. Only the sum of the total scores is presented in this study; total KOOS score was used as the criterion for evaluating function. The results from the final follow-up point were extracted in cases wherein the studies reported different or multiple post-injection time points. We combined data with similar follow-up periods because the studies of this meta-analysis had various follow-up periods for evaluation of clinical outcomes. Data from 4 and 6 weeks post-follow-up were merged with data from 1 month; data from 8 and 10 weeks were merged with 2 months; data from 16 and 18 weeks were merged with 4 months; and data from 26 weeks were merged with 6 months. We attempted to contact the study authors for supplementary information when there were insufficient or missing data in the articles.

### 2.4 Risk of bias assessment

Two reviewers independently assessed the risk of bias in the RCTs using the Cochrane Collaboration Risk of Bias tool.^[20]^ Each study was judged and scored as having high, low, or unclear risk of bias according to the following criteria: random sequence generation, allocation concealment, blinding of participants and personnel, blinding of outcome assessment, incomplete outcome data, selective reporting, and other biases. Disagreements between reviewers were resolved by discussion until consensus was obtained.

### 2.5 Statistical analyses

The main outcomes of the meta-analysis were the mean difference in VAS score change before and after injection for the PDRN and HA groups. Mean difference and 95% confidence intervals (CI) were calculated for continuous variables to compare primary outcomes (eg, pain VAS score). Secondary outcomes (KOOS, KSS score) were calculated and presented as standardized mean differences (SMDs) with 95% CIs depending on the different scales used for evaluation of function. Five studies included physical function assessments, and their data for score change from baseline were extracted.^[15,21–24]^ The relative risks (RRs) and 95% confidence intervals (CIs) were calculated for dichotomous outcome data (adverse events). Heterogeneity was determined using the *I*^2^ statistic, with values of 25%, 50%, and 75% considered as low, moderate, and high heterogenicity, respectively. If *I*^2^ <50%, pooled data were analyzed using a fixed-effects model; otherwise, a random-effects model was used. All statistical analyses were performed using RevMan version 5.3 (The Cochrane Collaboration, Copenhagen, Denmark).

## 3 Results

### 3.1 Identification of studies

**Figure 1:**
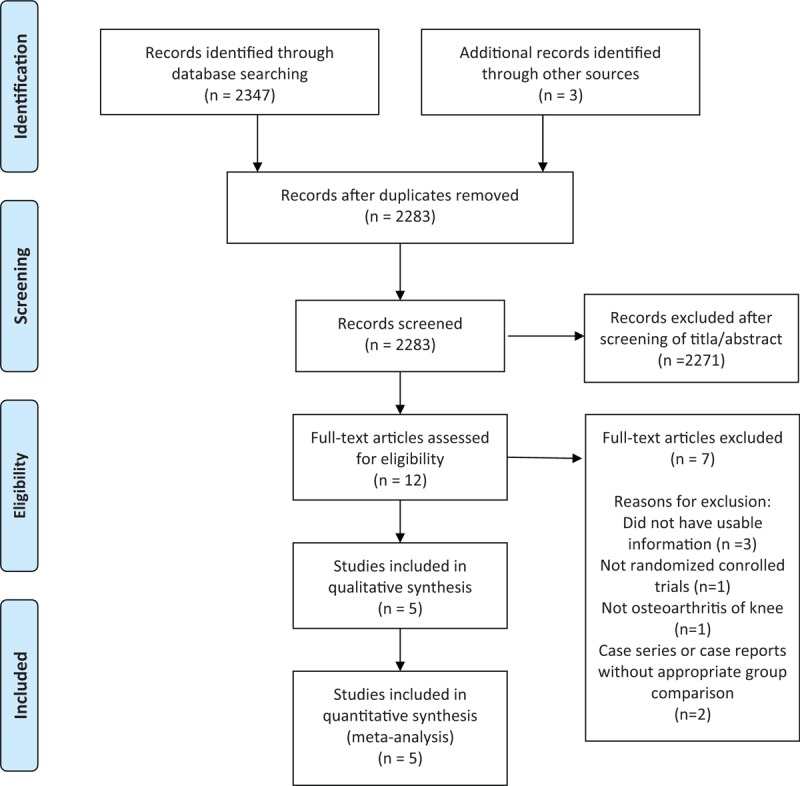
Flowchart illustrating the literature search.

A study flow diagram shows the process for study identification, inclusion, and exclusion (Fig. 1). An initial electronic search yielded 2283 studies. Three additional publications were obtained through manual searching. Twelve potentially eligible studies were assessed for inclusion after screening their titles and abstracts. After we reviewed the full texts, an additional seven studies were excluded. Finally, 5 RCTs were included in the meta-analysis.^[15,21–24]^

### 3.2 Study characteristics and quality of included studies

The study characteristics are summarized in Table 1.^[15,21–24]^ All studies were published from 2010 to 2018. There were more female patients than male patients (167 vs. 123). The number of patients per study ranged from 14 to 50, with a total 290 patients in the meta-analysis. Comparable patients were included in the PDRN group (145) and the HA group (145). One study had a 12-month follow-up point^[21]^; 3 studies had 6-month follow-up points^[21–23]^; 3 studies had 4-month follow-up points^[15,22,24]^; 4 studies had 2-month follow-up points^[15,21,22,24]^; and 4 studies had 1-month follow-up points.^[15,22–24]^

**Table 1:**
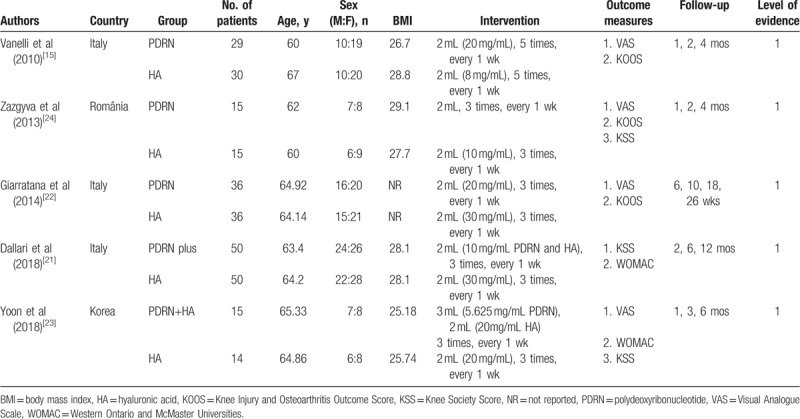
Characteristics of included studies.

### 3.3 Risk of bias

**Figure 2:**
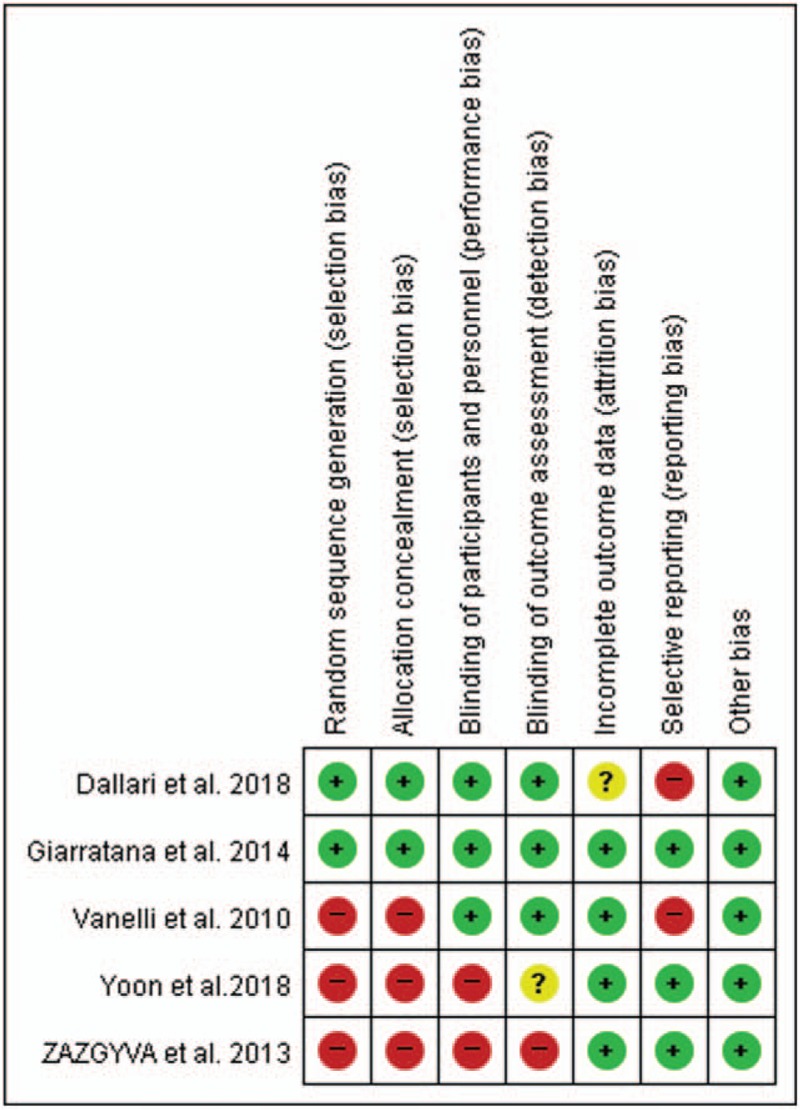
Risk of bias summary: review authors’ judgments for each risk of bias item for each included study. ? = unclear risk of bias, + = low risk of bias, − = high risk of bias.

Among the 5 studies, 2 had a low bias risk, and 3 had a high bias risk. An adequate randomized sequence was generated in 2 studies, appropriate allocation concealment was reported in 2 studies, blinding of participants was clearly present in 3 studies, and blinding of outcome assessors was reported in 3 studies. All methodological processes are shown in Figure 2.

### 3.4 PDRN versus HA

Pain VAS scores were reported in 4 studies among those that compared PDRN with HA.^[15,22,24]^ KOOS function and daily living, sport and recreation, pain, QOL, and symptoms were all reported in 2 studies^[15,22]^; KOOS total score was reported in 1 study^[24]^; KSS total score was reported in 3 studies^[21,24]^; and adverse events were reported in 5 studies.

### 3.5 The effect of PDRN injection on pain (VAS score)

**Figure 3:**
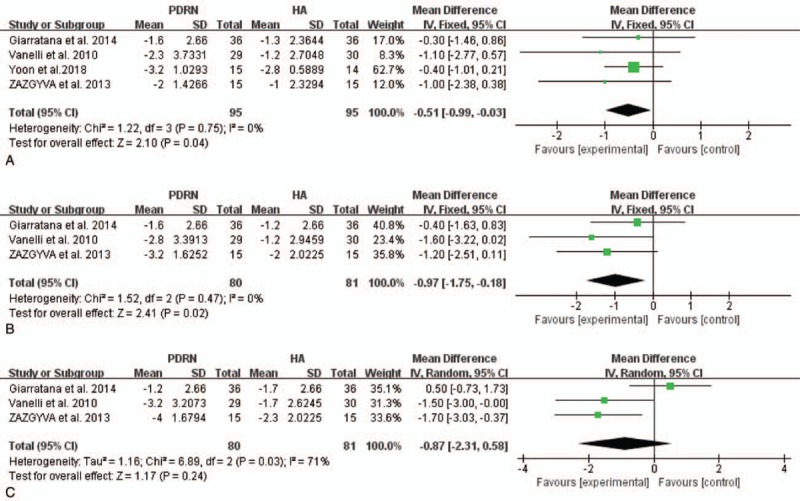
Forest plot of comparisons: PDRN versus HA for pain VAS score at 1 month (A), 2 months (B), and 4 months (C). CI = confidence interval, HA = hyaluronic acid, PDRN = polydeoxyribonucleotide, SD = standard deviation, VAS = Visual Analogue Scale.

Four studies at 1 month and 2 studies at 2 and 4 months assessed the effect of PDRN injection on pain using the VAS pain score. Our pooled analysis showed that patients treated with PDRN had less pain than those with HA (mean difference [MD] = −0.51, 95% confidence interval [CI]: −0.99 to 0.03, *P* = .04; Fig. 3) at 1 month with low heterogeneity (*I*^2^ = 0%). At 2 months, the data still showed significantly better pain improvement in the PDRN group compared with the HA group (MD = −0.97, 95% CI: −1.75– to 0.18, *P* = .02; Fig. 3) with low heterogeneity (*I*^2^ = 0%). At 4 months, there was no significant difference between the PDRN and HA groups (MD = −0.87, 95% CI: −2.31 to 0.58, *P* = .24; Fig. 3), and heterogeneity was significant in the pooled result (*I*^2^ = 71%).

### 3.6 The effect of PDRN injection on function

**Figure 4:**
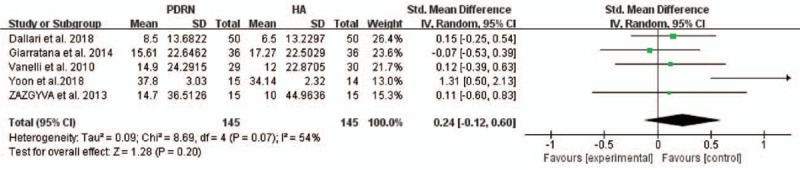
Forest plot of comparison: PDRN versus HA versus function (KOOS total and ADL scores, KSS total score). ADL = activity of daily living functions, CI = confidence interval, HA = hyaluronic acid, KOOS = Knee injury and Osteoarthritis Outcome Score, KSS = Knee Society Score, PDRN = polydeoxyribonucleotide, SD = standard deviation.

**Figure 5:**
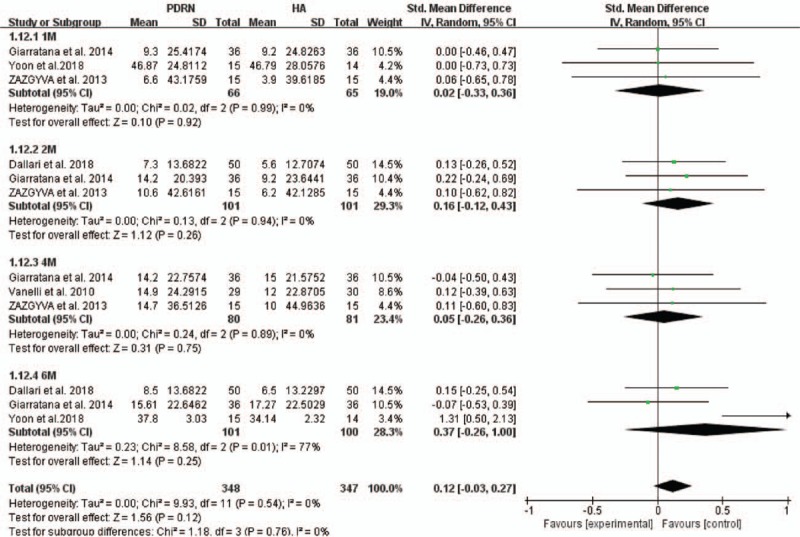
Subgroup meta-analyses of PDRN injection versus HA injection for function at 1, 2, 4, and 6 months. CI = confidence interval, HA = hyaluronic acid, PDRN = polydeoxyribonucleotide, SD = standard deviation.

Among all 5 studies, the KOOS score was used in 3. Two studies reported a score for 5 dimensions, and 1 study reported a total score. ADL was removed from the functional evaluations. The pooled analysis showed that no significant difference was seen in function scores (KOOS ADL score, KOOS total score, and KSS score) between the PDRN and HA groups (SMD (CI) = 0.24 (−0.12–0.60), *P* = .20; Fig. 4). Heterogeneity was moderate in the pooled result (*I*^2^ = 54%). Additionally, subgroup analyses were performed for each follow-up point. The results showed no significant difference in functional outcomes between the 2 groups at 1 month (SMD = 0.02, 95% CI: −0.33 to 0.36, *P* = .92), 2 months (SMD = 0.16, 95% CI: −0.12 to 0.43, *P* = .26), 4 months (SMD = 0.05, 95% CI: −0.26 to 0.36, *P* = .75), and 6 months (SMD = 0.37, 95% CI: −0.26 to 1.00, *P* = .25; Fig. 5). Heterogeneity was present at all time points (*I*^2^ = 0% at 1, 2, 4 months and *I*^2^ = 77% at 6 months).

### 3.7 Adverse events

Five studies compared the risk of adverse events. We found no significant difference between the PDRN and HA groups in terms of adverse events (RR = 2.15, 95% CI: 0.17–26.67, *P* = .55).

## 4 Discussion

Intra-articular injection of PDRN provided significant pain reduction 1 and 2 months after knee OA compared with HA. Functional improvement after injection was comparable to HA. The incidence of attributable adverse events was low in the PDRN and HA groups, and the difference in events between the groups was not significant.

Polynucleotides display trophic activity combined with a mixture of purines, pyrimidines, deoxyribonucleotides, and deoxyribonucleosides.^[25]^ The compound has a viscoelastic property as a result of how it interacts with water.^[25–27]^ Polynucleotides are also associated with induced cell growth, collagen production, migration of several cell types, and reduced inflammation.^[25–27]^ In-vitro and in-vivo studies have verified the effect of PDRN on cartilage.^[16,25]^ Bitto et al^[25]^ reported that PDRN injections improved arthritis symptoms, decreased the expression of proinflammatory factors, such as high-mobility group protein-1, tumor necrosis factor-α, and interleukin (IL)-6, and increased anti-inflammatory IL-10 expression using a collagen-induced arthritis animal model. PDRNs are suitable for cultivation of in-vitro cartilage and have displayed a therapeutic effect on chondrocytes by protecting cartilage.^[16]^ Based on this knowledge, studies of intra-articular PDRN injections to treat knee OA have been carried out.

Clinical trials on actual patients are necessary to establish and confirm the efficacy and value of new treatments. Recent studies have shown that intra-articular injection of PDRN helps reduce pain and improves function in patients with knee OA.^[15,21–24]^ However, there are still no large-scale RCTs involving many patients^[15,21–24]^; thus, the effect of PDRN injection on knee OA remains unclear because the number of study patients is small.^[15,21–24]^ This meta-analysis, which included 5 previously published RCTs, is the first to evaluate the effect of PDRN injection on patients with knee OA. The strengths of this study include comprehensive and transparent search strategies, independent and duplicate qualification assessment, and data extraction, and the use of standard meta-analysis techniques to evaluate the effectiveness of PDRN injection for knee OA. The 5 RCTs we assessed to evaluate PDRN injection for knee OA treatment met the requirements for systematic review.

Four of 5 studies evaluated postinjection pain using a VAS pain assessment. Pain was measured at several time points after injection. At 1 and 2 months post-injection, the PDRN group experienced greater pain reduction than the HA group, but at 4 and 6 months, there was no difference between the 2 groups. Although there was no difference in pain relief between the 2 groups after 4 months, our study confirmed an advantage of PDRN over HA for pain reduction through the second month post-injection.

This study found greater efficacy of PDRN compared with the control group who only received HA. Improvement in knee function after HA treatment for OA has already been demonstrated by many studies.^[8]^ All the included studies in this meta-analysis evaluated knee function in OA patients after PDRN injection.^[15,21–24]^ However, because these studies used various evaluation tools and measurement points, the last measurement point was set as the evaluation point, and KOOS and KSS scores were used for our functional evaluations. Overall, there was no significant positive effect of PDRN injection compared with HA. In a subgroup analysis for more robust estimates, we also found that PDRN showed similar efficacy to HA with regard to function improvement at 1, 2, 4, and 6 months.

The American Academy of Orthopaedic Surgeons (AAOS),^[28]^ Osteoarthritis Research Society International (OARSI),^[29]^ and American College of Rheumatology (ACR)^[30]^ guidelines suggest that HA is either not recommended or yields inconclusive results. However, HA injection has been recently shown by several studies to be safer and less adverse for the treatment of knee OA.^[8,31–34]^ All 5 studies included in this meta-analysis investigated the adverse events of PDRN injections for knee OA compared with HA injection. All 5 studies reported few side effects, and there were no significant differences in adverse events between the PDRN group and the HA group. In the case of PDRN injection, no particular side effects have been identified.

Our study has several limitations. First, there is substantial heterogeneity in patient characteristics in our meta-analysis studies with regard to patient age, sex, body-mass index, and OA grade. These factors could potentially differently influence patient physiological responses. Second, the studies included in the meta-analysis had significant methodological limitations. The potential risk of bias from these studies weakened our inference about the therapeutic effects of PDRN injection for knee OA. Third, the physical function measures were different across the studies in the meta-analysis. We compared functional outcomes between the PDRN and HA groups mainly using the KOOS and the total KSS scores. It is important to consider how results are measured when evaluating the effect of PDRN injection on knee OA. If the outcome measurement for function is not variable and integrated into a single measure, a more accurate and clearer measurement should be attempted. Fourth, our meta-analysis included a limited number of small-scale clinical trials—5 studies with a total of 290 participants; thus, we could not determine the risk of publication bias. It is possible that only positive clinical trials get published, whereas negative clinical trials get rejected for publication. All these methodological limitations reduced the reliability of the observed effect estimates in our meta-analyses, and a type-II statistical error owing to an underpowered analysis could have occurred. Thus, larger RCTs with long-term follow-up are needed to truly understand the effects of PDRN on knee OA. Finally, we included only articles published in English, which could have introduced a language or cultural bias.

## 5 Conclusion

The intra-articular use of PDRN yielded similar functional outcomes to HA, and the pain relief effect was superior for up to 2 months post-injection. Therefore, PDRN could be a favorable alternative to HA for treating knee OA with persistent pain, while also avoiding common HA side effects. Additionally, the pain-relief benefits of PDRN in clinical practice could offer a complementary role for other injection treatments.

## Author contributions

**Conceptualization:** Man Soo Kim.

**Data curation:** Man Soo Kim, Ryu Kyung Cho.

**Formal analysis:** Man Soo Kim.

**Investigation:** Man Soo Kim, Ryu Kyung Cho.

**Methodology:** Man Soo Kim, Ryu Kyung Cho.

**Software:** Man Soo Kim, Ryu Kyung Cho.

**Supervision:** Yong In.

**Validation:** Yong In.

**Writing – original draft:** Man Soo Kim.

**Writing – review & editing:** Yong In.

Yong In orcid: 0000-0002-5932-3934.
